# Synthesis and crystal structure of 5,10-dihy­droxy-9-meth­oxy-2,2-dimethyl-12-(2-methyl­but-3-en-2-yl)-2*H*,6*H*-pyrano[3,2-*b*]xanthen-6-one

**DOI:** 10.1107/S2056989025001070

**Published:** 2025-02-14

**Authors:** Amporn Saekee, Chutima Kuhakarn, Khetpakorn Chakarawet, Sakchai Hongthong

**Affiliations:** a Department of Chemistry and Center of Excellence for Innovation in Chemistry, (PERCH-CIC), Faculty of Science, Mahidol University, Bangkok 10400, Thailand; b Department of Chemistry, Faculty of Science, Mahidol University, Bangkok 10400, Thailand; c Program in Chemistry Faculty of Science and Technology Rajabhat Rajanagarindra University,Chachoengsao 24000 Thailand; Indian Institute of Science Education and Research Bhopal, India

**Keywords:** crystal structure, xanthone, natural products

## Abstract

5,10-Dihy­droxy-9-meth­oxy-2,2-dimethyl-12-(2-methyl­but-3-en-2-yl)-2*H*,6*H*-pyrano[3,2-*b*]xanthen-6-one, C_24_H_24_O_6_ (**2**), is a prenylated xanthone that was synthesized from 5,9,10-trihy­droxy-2,2-dimethyl-12-(2-methyl­but-3-en-2-yl)-2*H*,6*H*-pyrano[3,2-*b*]xanthen-6-one or macluraxanthone (**1**), a known compound isolated from *Garcinia schomburgkiana* Pierre. The present study describes the synthesis of compound **2** by methyl­ation reaction of **1**, and its crystallographic characterization. Compound 2 features a planar xanthone core and a bent pyrano ring adopting a half-boat conformation. An intermolecular O—H⋯O hydrogen bond between the hydroxyl hydrogen donor and the ketone acceptor organizes the molecules into a one-dimensional network along the *b*-axis direction. Perpendicular to this network, π–π stacking interactions form the three-dimensional supramolecular architecture. These two key intermolecular interactions are distinctly revealed in the Hirshfeld surface analysis.

## Abstract 2

Methyl­ation of the natural product macluraxanthone yielded its meth­oxy analog, which was characterized by a suite of spectroscopic and crystallographic techniques.

## 1. Chemical context

Pyran­oxanthones have long been known for their natural occurrence, showing a broad spectrum of pharmacological and biological activities (Kondedeshmukh & Paradkar, 1994 ▸). In the past decade, this scaffold has been subjected to chemical structure identification and synthetic investigations. The pyran­oxanthone core also brings a wide range of applications. For example, 1,2-di­hydro-2-hy­droxy-6-meth­oxy-3,3-dimethyl-3*H*,7*H*-pyrano[2,3-*c*]xanthen-7-one showed a potent cytotoxicity against leukemia L1210 cell line (Ghirtis *et al.*, 2001 ▸). Because various substituents on pyran­oxanthones cause different properties, the structure–activity relationships (SAR) play a pivotal role in the discovery of their biological activities.

**Figure 1:**
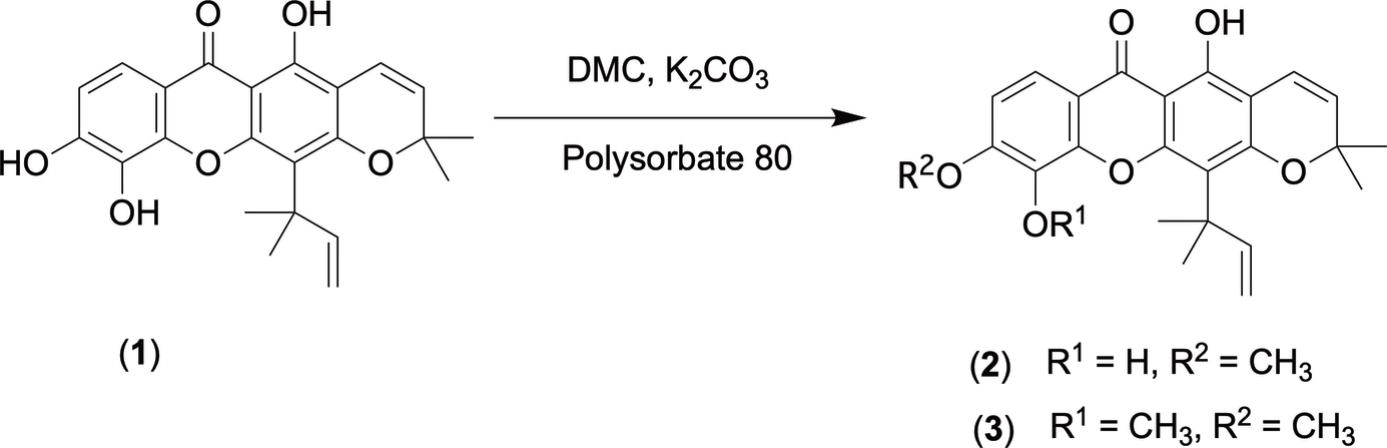
Methyl­ation reaction of macluraxanthone (**1**) to produce xanthones **2** and **3**.

5,10-Dihy­droxy-9-meth­oxy-2,2-dimethyl-12-(2-methyl­but-3-en-2-yl)-2*H*,6*H*-pyrano[3,2-*b*]xanthen-6-one (**2**) is a pyran­oxanthone that was isolated from leaves and twigs of *Garcinia speciosa*. This compound showed weak inhibitory activity toward HIV-1 reverse transcriptase (Pailee *et al.*, 2018 ▸). In addition, a parent analog 5,9,10-trihy­droxy-2,2-dimethyl-12-(2-methyl­but-3-en-2-yl)-2*H*,6*H*-pyrano[3,2-*b*]xanthen-6-one (**1**), also known as macluraxanthone, was isolated from the same plant (Sangsuwon & Jiratchariyakul, 2015 ▸). Compound **1** was first isolated from osage orange (*Maclura pomifera*) in 1964 (Wolfrom *et al.*, 1964 ▸). Subsequently, it was also found in different parts of various plants such as *Garcinia bancana* (Rifaldi *et al.*, 2024 ▸), and *Cratoxylum soulattri* (Mah *et al.*, 2011 ▸). Recently, **1** was also isolated from fruits and twigs of *Garcinia schomburgkiana*, a Thai plant known locally as ‘Ma Dun’, with a considerable qu­antity (1.07% from fruits and 5.29% from twigs) (Sukkum *et al.*, 2024 ▸). Prompted by the above results, we performed the synthesis of compounds **2** and **3** from macluraxanthone (**1**) *via* methyl­ation reaction using dimethyl carbonate (DMC) as a methyl­ating reagent in the presence of K_2_CO_3_ as a base, and polysorbate 80 as a phase transfer catalyst (Prakoso *et al.*, 2016 ▸) as shown in Fig. 1 ▸. The identities of the products were confirmed by ^1^H and ^13^C nuclear magnetic resonance (NMR) spectroscopy and high-resolution electrospray ionization mass spectrometry (HR-ESI-MS). Based on the spectroscopic and spectrometry data and by comparison with the data reported in the literature (Pailee *et al.*, 2018 ▸; Wolfrom *et al.*, 1964 ▸), compounds **2** and **3** were determined as 5,10-dihy­droxy-9-meth­oxy-2,2-dimethyl-12-(2-methyl­but-3-en-2-yl)-2*H*,6*H*-pyrano[3,2-*b*]xanthen-6-one (**2**) and 5-hy­droxy-9,10-dimeth­oxy-2,2-dimethyl-12-(2-methyl­but-3-en-2-yl)-2*H*, 6*H*-pyrano[3,2-*b*]xanthen-6-one (**3**). The structure of **2** was further confirmed by single-crystal X-ray crystallography.
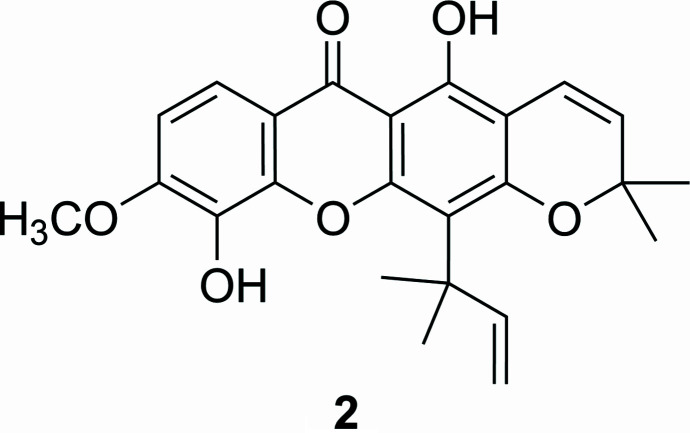


## 2. Structural commentary

**Figure 2:**
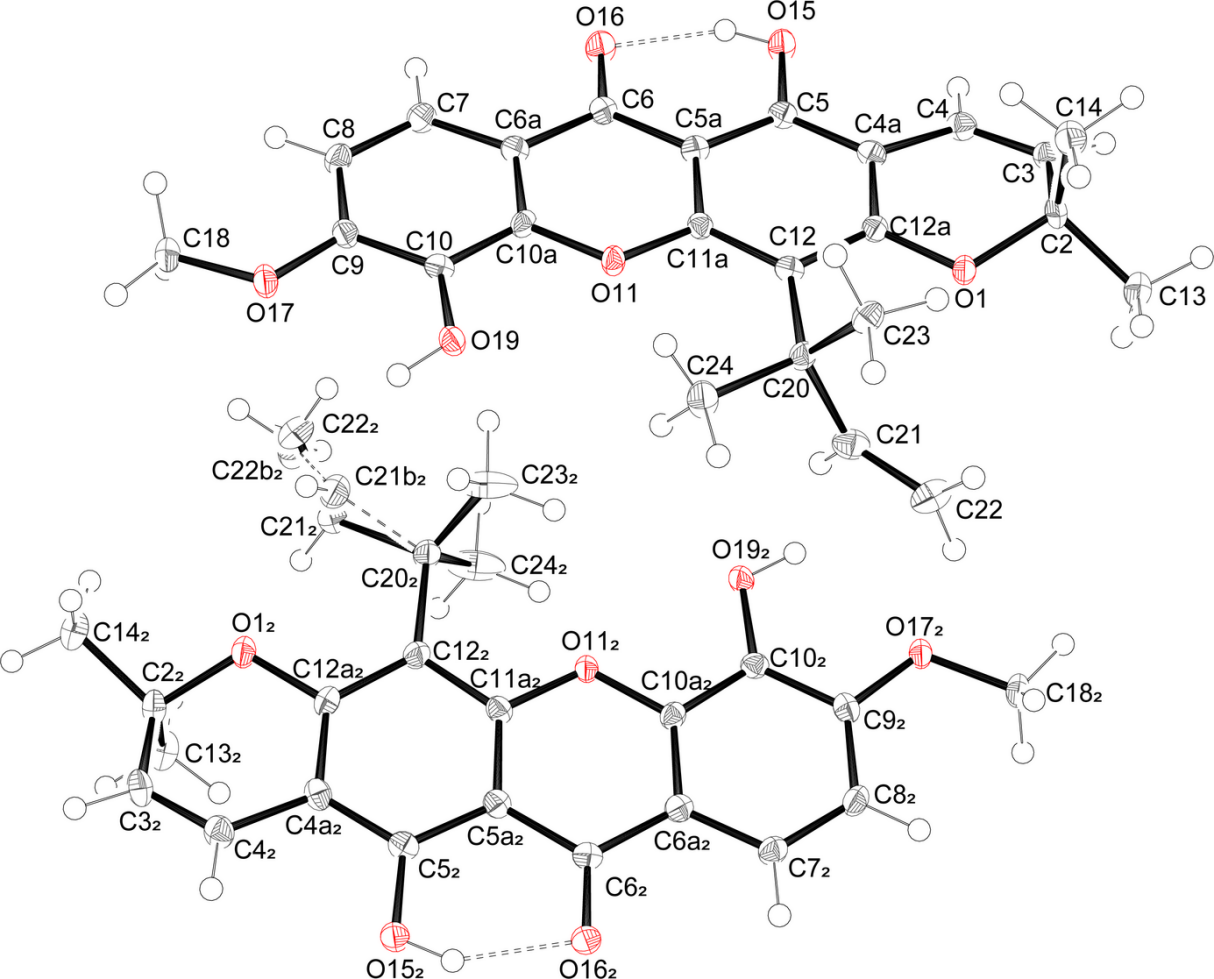
*ORTEP* view of compound **2** plotted as displacement ellipsoids at the 50% probability level. Two mol­ecules comprise the asymmetric unit.

Compound **2** crystallizes in the monoclinic *P*2_1_ space group with two independent mol­ecules in the asymmetric unit (*Z* = 4). The structure of **2** shows the expected methyl­ation of the hydroxyl group at O17 position (Fig. 2 ▸). The methyl group is coplanar with the core xanthone structure, with C8—C9—O17—C18 torsion angles of 3.8 (3) and −1.4 (3)° for the first and second mol­ecules, respectively. The xanthone core structure (O11, C4*A*–C12*A*) is planar, while the pyrano ring (O1, C2–C4*A*, C12*A*) is bent, adopting a half-boat conformation with the C2 atom deviating out of the plane generated by the remaining 17 atoms (O1, O11, C3–C12*A*) by 0.456 (2) and 0.534 (2) Å, for the first and second mol­ecules, respectively. This result supports the chemical structure that C2 is not conjugated with the aromatic system. The root-mean-square deviations of the mol­ecular plane formed from these 17 atoms are 0.034 and 0.035 Å for the first and second mol­ecules, respectively.

The structure of **2** features an intra­molecular hydrogen bond (Table 1 ▸) between its carbonyl and the nearby hydroxyl group, with an O15⋯O16 distance of 2.530 (2) and 2.547 (2) Å for the first and second mol­ecules in the asymmetric unit, respectively. This distance is considered relatively short for O⋯O distances involved in hydrogen bonding.

## 3. Supra­molecular features

**Figure 3:**
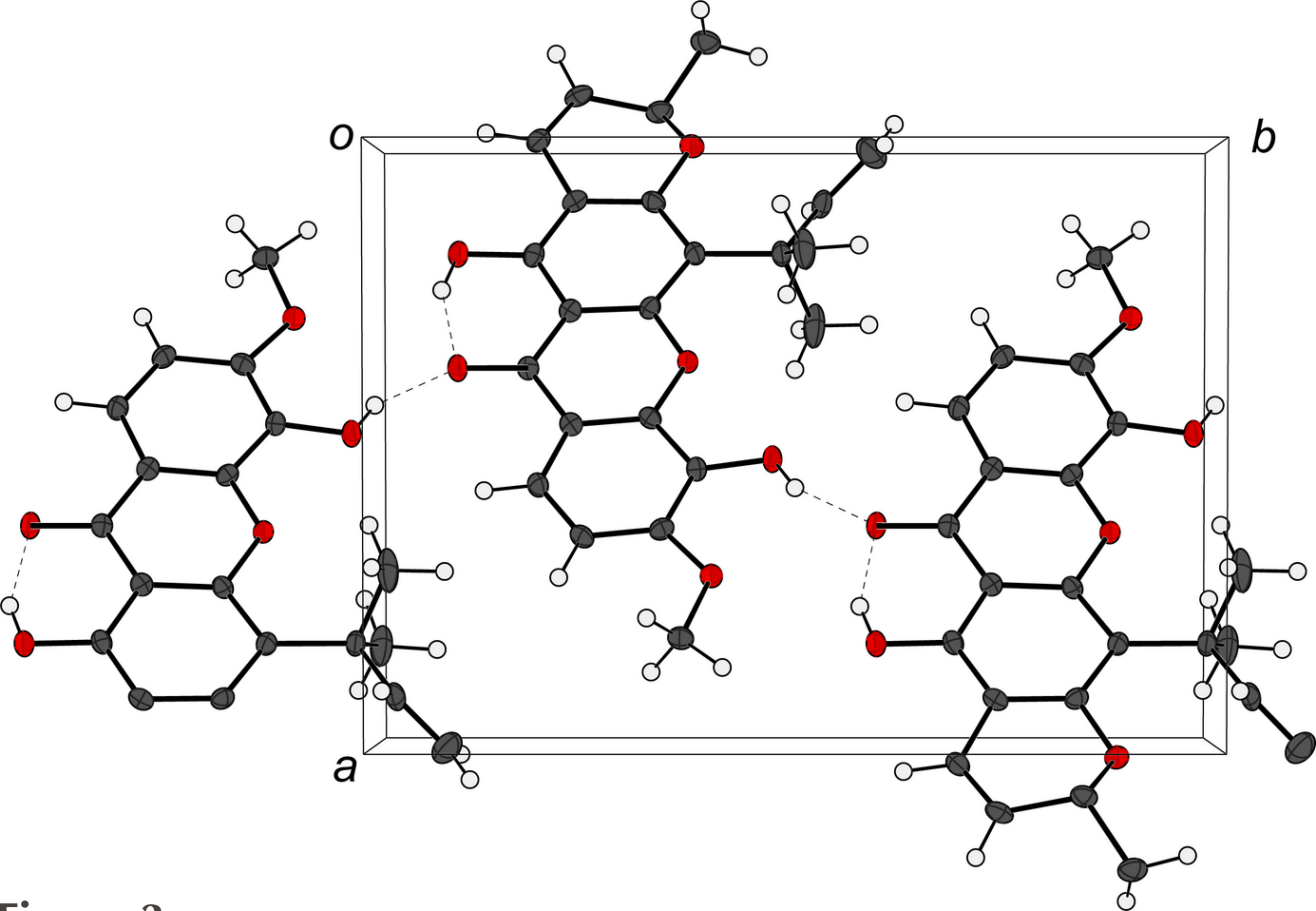
Packing of **2** in the unit cell, consolidated by inter­molecular hydrogen bonding. The unit cell is shown as a gray box where the *c* axis is parallel to the reader’s view. The *a* and *b* axes and the origin center are labeled in the figure.

An inter­molecular hydrogen bond is found between O16 and O19 with an O⋯O distance of 2.719 (2) and 2.704 (2) Å (Table 1 ▸) for the first and second mol­ecules in the asymmetric unit, respectively. This hydrogen bonding consolidates the mol­ecular packing, which forms a one-dimensional network of **2** along the *b*-axis direction (Fig. 3 ▸). The meth­oxy group of **2** formed upon the methyl­ation of **1** is not involved in any significant hydrogen-bonding inter­actions.

**Figure 4:**
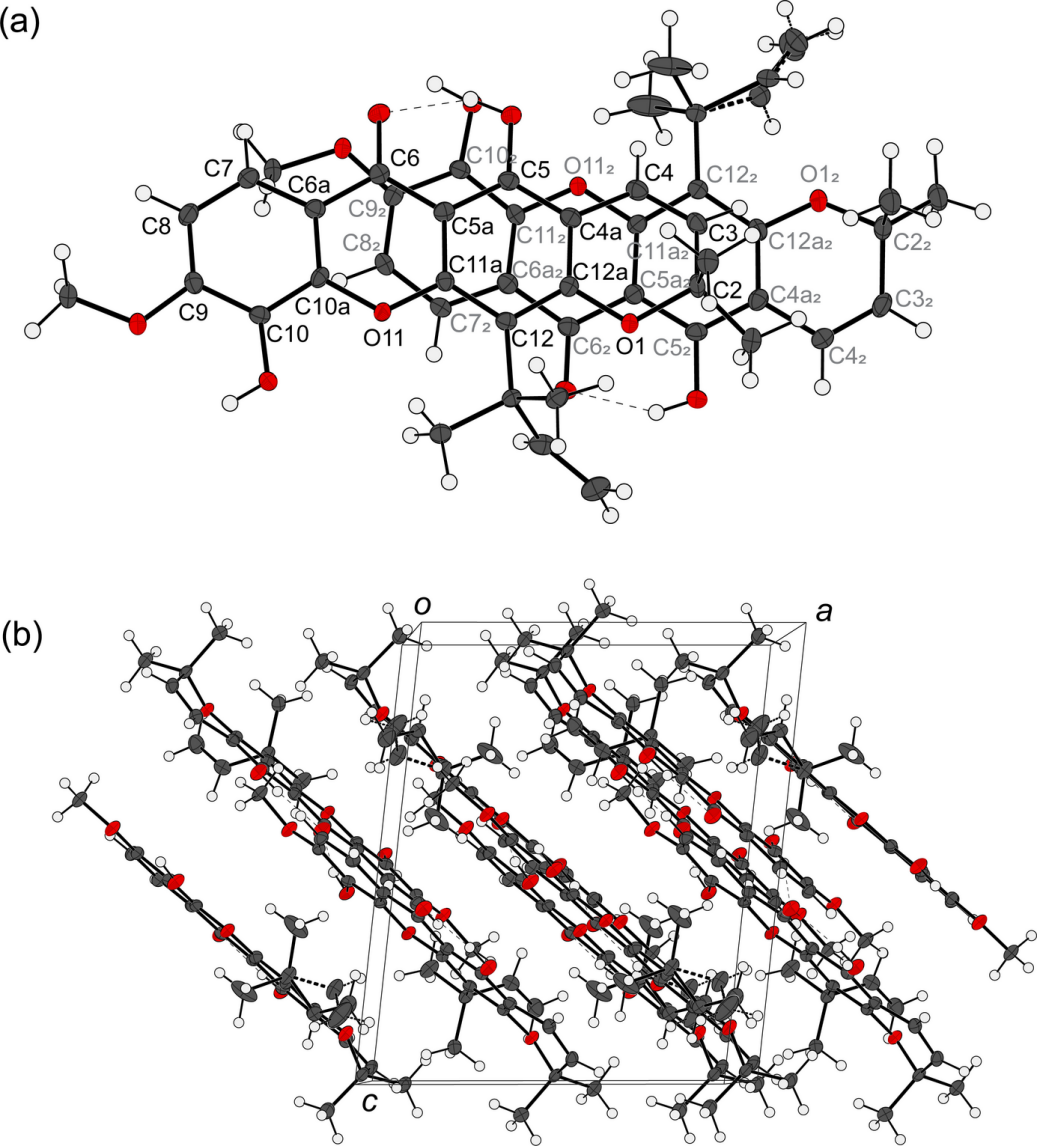
(*a*) Two mol­ecules of **2** in the asymmetric unit viewed perpendicular to the pyran­oxanthone rings to highlight π–π stacking. The pyran­oxanthone core atoms of the first and second mol­ecules are labeled in black and gray, respectively. (*b*) Stacking of **2** in the unit cell. The unit cell is shown as a gray box where the *b* axis is parallel to the reader’s view. The *a* and *c* axes and the origin center are labeled in the figure.

The planarity of the mol­ecule facilitates mol­ecular stacking in the structure. The first and second mol­ecules in the asymmetric unit are aligned almost parallel to each other; the angle between the two mol­ecular planes are 11.23 (4)°. The shortest C⋯C distance between the two mol­ecules is 3.366 (3) Å, found between C4 of the first mol­ecule and C11*A* of the second mol­ecule, indicating possible π–π inter­actions (Fig. 4 ▸*a*). The π–π stackings run along the [10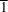] direction perpendicular to the inter­molecular hydrogen-bonding network (Fig. 4 ▸*b*), altogether forming a three-dimensional supramolecular arrangement.

## 4. Hirshfeld surface analysis

**Figure 5:**
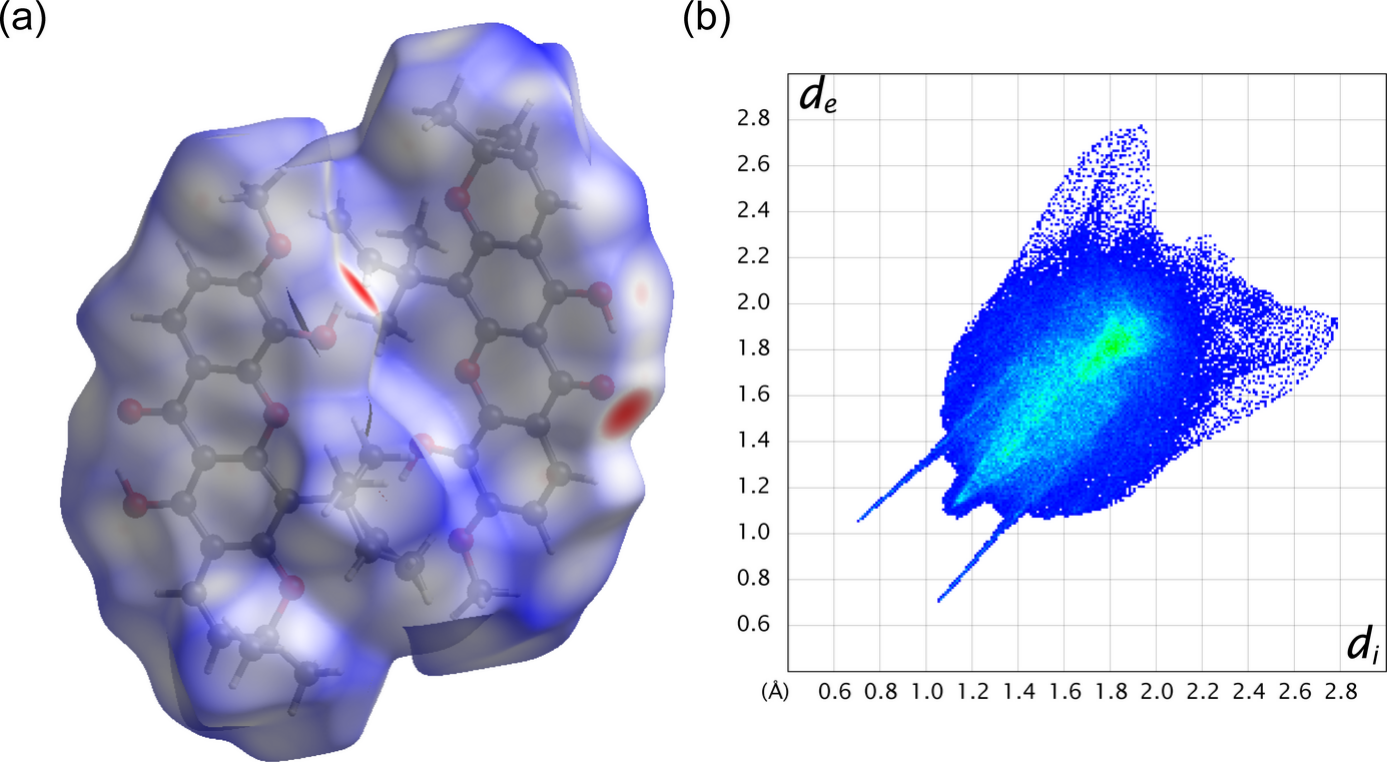
(*a*) Three-dimensional Hirshfeld surface representation of **2** plotted over *d*_norm_ and (*b*) two-dimensional fingerprint plot of **2** showing all inter­actions.

Hirshfeld surface analysis was performed to more accurately identify and qu­antify inter­molecular inter­actions. The analysis was performed using *CrystalExplorer* 21.5 (Spackman *et al.*, 2021 ▸). The three-dimensional Hirshfeld surface of **2** is plotted in Fig. 5 ▸*a*, including the two mol­ecules of the asymmetric unit, mapped over normalized contact distance (*d*_norm_) on a scale from −0.65 to 1.66 a.u. Blue, white, and red regions indicate contacts that are longer, equal, and shorter than the sum of van der Waals radii, respectively. The apparent red regions around O atoms indicate short contacts from inter­molecular hydrogen bonding. Fig. 5 ▸*b* depicts a two-dimensional fingerprint plot of (*d*_i_, *d*_e_). Two sharp spikes in the fingerprint plot indicate the short inter­molecular O—H⋯O hydrogen bonding inter­actions, which contribute 15.8% to the overall Hirshfeld surface area. A π–π planar stacking was also identified in the cyan-green region of the plot centered around *d*_i_ = *d*_e_ = 1.8 Å, contributing 8.7%. The surface corresponding to these C⋯C inter­actions spans over two of the four aromatic rings of **2**, indicating that π–π stacking is impeded. The remaining major inter­molecular inter­actions are H⋯H and C⋯H inter­actions, contributing 62.4 and 9.7%, respectively.

## 5. Database survey

A search for the pyran­oxanthone core structure revealed five crystal structures in the Cambridge Structural Database (CSD version 5.45, last update November 2023; Groom *et al.*, 2016 ▸). The structure of macluraxanthone (**1**) (QAYTOA; Fun *et al.*, 2006 ▸) shows similar structural features including the bent pyrano ring and planar xanthone rings, but differs in the inter­molecular packing: **1** engages in hydrogen-bonding inter­actions involving the O17 position, which is absent in **2** due to the methyl­ation of this oxygen atom. A di-*p*-bromo­benzene­sulfonyl­ated derivative was also reported (YIZPAZ; Boonnak *et al.*, 2008 ▸), featuring a similar bent pyrano ring and planar xanthone rings. In addition, three other pyran­oxanthone structures possessing different substituents on C12 were found in the database: CIXSIL (Kosela *et al.*, 1999 ▸), MAPMIA (Chantrapromma *et al.*, 2005 ▸), and CABFAP (Sukandar *et al.*, 2016 ▸).

## 6. Synthesis and crystallization

The synthetic reaction was modified from a published procedure according to Prakoso *et al.* (2016 ▸). Briefly, a 50 mL round-bottom flask equipped with a magnetic stir bar was charged with **1** (0.13 mmol, 1 equiv.), K_2_CO_3_ (0.56 mmol, 4.3 equiv.), and polysorbate 80 (0.163 mmol, 1.25 equiv.). Then, dimethyl carbonate (DMC) (1.30 mmol, 10 equiv.) was added to the reaction mixture. After refluxing at 373 K for 5 h, the reaction mixture was quenched with aqueous acetic acid (20 mL) and extracted with di­chloro­methane (5 × 50 mL). The combined organic layers were washed with a saturated NaCl solution (20 mL) and dried over anhydrous Na_2_SO_4_. After removal of the solvent, the crude mixture was purified by column chromatography (acetone:hexane, 1:5 *v*/*v*, isocratic system) to afford compound **2** (48% yield), compound **3** (11% yield), and a recovered starting material (**1**) (36% yield).

5,10-Dihy­droxy-9-meth­oxy-2,2-dimethyl-12-(2-methyl­but-3-en-2-yl)-2*H*,6*H*-pyrano[3,2-*b*]xanthen-6-one (**2**): yellow solid, ^1^H NMR (400 MHz, CDCl_3_), *δ* 13.48 (*s*, 1H), 7.68 (*d*, *J* = 9.0 Hz,1H), 6.90 (*d*, *J* = 9.0 Hz, 1H), 6.69 (*d*, *J* = 10.0 Hz, 1H), 6.61 (*s*, 1H), 6.59 (*dd*, *J* = 16.0, 10.0 Hz, 1H), 5.54 (*d*, *J* = 10.0 Hz, 1H), 5.12 (*dd*, *J* = 16.0, 1.0 Hz, 1H), 5.09 (*dd*, *J* = 10.0, 1.0 Hz, 1H), 3.95 (*s*, 3H), 1.59 (*s*, 6H), 1.44 (*s*, 6H) ppm. ^13^C NMR (100 MHz, CDCl_3_), *δ* 180.8, 159.1, 156.7, 154.9, 154.4, 151.5, 144.3, 133.5, 127.1, 116.8, 116.0, 114.2, 113.3, 108.4, 105.4, 104.6, 103.6, 78.2, 55.6, 41.3, 28.5, 27.1 ppm. HR-ESI-MS of 407.1482 [*M*–H]^−^ (calculated for C_24_H_23_O_6_; 407.1500).

5-Hy­droxy-9,10-dimeth­oxy-2,2-dimethyl-12-(2-methyl­but-3-en-2-yl)-2*H*,6*H*-pyrano[3,2-*b*]xanthen-6-one (**3**): yellow solid, ^1^H NMR (400 MHz, CDCl_3_), *δ* 13.57 (*s*, 1H, OH), 7.90 (*d*, *J* = 8.0 Hz, 1H), 6.92 (*d*, *J* = 8.0 Hz, 1H), 6.68 (*d*, *J* = 9.6 Hz, 1H), 6.30 (*dd*, *J* = 12.1, 8.0 Hz, 1H), 5.52 (*d*, *J* = 9.6 Hz, 1H), 4.86 (*dd*, *J* = 17.0, 1.0 Hz, 1H), 4.78 (*dd*, *J* = 10.0, 1.0 Hz, 1H), 3.94 (*s*, 3H, OCH_3_), 3.86 (*s*, 3H, OCH_3_), 1.66 (*s*, 6H), 1.40 (*s*, 6H) ppm. ^13^C NMR (100 MHz, CDCl_3_), *δ* 181.0, 159.4, 158.0, 156.5, 155.4, 150.8, 150.0, 136.4, 127.3, 121.4, 116.0, 114.9, 113.7, 108.6, 107.9, 105.2, 103.3, 78.2, 61.5, 55.4, 41.5, 31.9, 27.9 ppm. HR-ESI-MS of 445.1622 [*M* + Na]^+^ (calculated for C_25_H_26_O_6_Na; 445.1622).

Single crystals of **2** were obtained as yellow blocks from vapor diffusion of *n*-hexane into an acetone solution.

## 7. Refinement

Crystal data, data collection and structure refinement details are summarized in Table 2 ▸. All non-hydrogen atoms were refined anisotropically. The vinyl group of the second mol­ecule was found to be disordered; refinement was accomplished by modeling over two positions. The occupancy of each disordered component was initially refined freely, and converged to a 0.53:0.47 occupancy ratio. Thus, the occupancy of both components was subsequently constrained to 0.5. The anisotropic displacement refinement of the disordered atoms was stabilized by the application of enhanced rigid bond restraints. Hydrogen atoms bonded to carbon were included in calculated positions and refined using a riding model. Hydrogen atoms bound to O atoms were located in the difference-Fourier map, and refined semi-freely with the help of distance restraints.

## Supplementary Material

Crystal structure: contains datablock(s) I. DOI: 10.1107/S2056989025001070/dx2063sup1.cif

Structure factors: contains datablock(s) I. DOI: 10.1107/S2056989025001070/dx2063Isup2.hkl

Supporting information file. DOI: 10.1107/S2056989025001070/dx2063Isup3.mol

Supporting information file. DOI: 10.1107/S2056989025001070/dx2063Isup4.cml

CCDC reference: 2421853

Additional supporting information:  crystallographic information; 3D view; checkCIF report

## Figures and Tables

**Table 1:** Hydrogen-bond geometry (Å, °)

*D*—H⋯*A*	*D*—H	H⋯*A*	*D*⋯*A*	*D*—H⋯*A*
O15—H15⋯O16	0.84	1.78	2.530 (2)	147
O15_2—H15_2⋯O16_2	0.84	1.80	2.547 (2)	147
O19—H19⋯O16^i^	0.84	1.92	2.719 (2)	159
O19_2—H19_2⋯O16_2^ii^	0.84	1.92	2.704 (2)	156

Symmetry codes: (i) 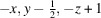; (ii) 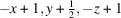.

**Table 2:** Experimental details

Crystal data	
Chemical formula	C_24_H_24_O_6_
*M*_r_	408.43
Crystal system, space group	Monoclinic, *P*2_1_
Temperature (K)	101
*a*, *b*, *c* (Å)	10.6205 (3), 14.8160 (4), 12.7073 (3)
β (°)	96.209 (1)
*V* (Å^3^)	1987.81 (9)
*Z*	4
Radiation type	Cu *K*α
μ (mm^−1^)	0.80
Crystal size (mm)	0.18 × 0.11 × 0.06

Data collection	
Diffractometer	Bruker D8 QUEST PHOTON III C7
Absorption correction	Multi-scan (*SADABS*; Krause *et al.*, 2015 ▸)
*T*_min_, *T*_max_	0.703, 0.753
No. of measured, independent and observed [*I* > 2σ(*I*)] reflections	32650, 7256, 6891
*R*_int_	0.042
(sin θ/λ)_max_ (Å^−1^)	0.604

Refinement	
*R*[*F*^2^ > 2σ(*F*^2^)], *wR*(*F*^2^), *S*	0.031, 0.079, 1.06
No. of reflections	7256
No. of parameters	581
No. of restraints	253
H-atom treatment	H atoms treated by a mixture of independent and constrained refinement
Δρ_max_, Δρ_min_ (e Å^−3^)	0.20, −0.25
Absolute structure	Flack *x* determined using 3110 quotients [(*I*^+^)−(*I*^−^)]/[(*I*^+^)+(*I*^−^)] (Parsons *et al.*, 2013 ▸)
Absolute structure parameter	0.00 (5)

Computer programs: *APEX2* and *SAINT* (Bruker, 2016 ▸), *SHELXT2015* (Sheldrick, 2015*a* ▸), *SHELXL* (Sheldrick, 2015*b* ▸), *ORTEP-3 for Windows* (Farrugia, 2012 ▸) *OLEX2* (Dolomanov *et al.*, 2009 ▸) and *publCIF* (Westrip, 2010 ▸).

## supplementary crystallographic information

### 5,10-Dihydroxy-9-methoxy-2,2-dimethyl-12-(2-methylbut-3-en-2-yl)-2H,6H-pyrano[3,2-b]xanthen-6-one Crystal data

**Table:**

C_24_H_24_O_6_	*F*(000) = 864
*M**_r_* = 408.43	*D*_x_ = 1.365 Mg m^−^^3^
Monoclinic, *P*2_1_	Cu *K*α radiation, λ = 1.54178 Å
*a* = 10.6205 (3) Å	Cell parameters from 9895 reflections
*b* = 14.8160 (4) Å	θ = 4.6–68.5°
*c* = 12.7073 (3) Å	µ = 0.80 mm^−^^1^
β = 96.209 (1)°	*T* = 101 K
*V* = 1987.81 (9) Å^3^	Block, clear light yellow
*Z* = 4	0.18 × 0.11 × 0.06 mm

### 5,10-Dihydroxy-9-methoxy-2,2-dimethyl-12-(2-methylbut-3-en-2-yl)-2H,6H-pyrano[3,2-b]xanthen-6-one Data collection

**Table:**

Bruker D8 QUEST PHOTON III C7 diffractometer	6891 reflections with *I* > 2σ(*I*)
Radiation source: microfocus sealed X-ray tube, Incoatec Iµs	*R*_int_ = 0.042
ω and φ scans	θ_max_ = 68.6°, θ_min_ = 4.2°
Absorption correction: multi-scan (SADABS; Krause *et al.*, 2015)	*h* = −11→12
*T*_min_ = 0.703, *T*_max_ = 0.753	*k* = −17→17
32650 measured reflections	*l* = −15→15
7256 independent reflections	

### 5,10-Dihydroxy-9-methoxy-2,2-dimethyl-12-(2-methylbut-3-en-2-yl)-2H,6H-pyrano[3,2-b]xanthen-6-one Refinement

**Table:**

Refinement on *F*^2^	Hydrogen site location: mixed
Least-squares matrix: full	H atoms treated by a mixture of independent and constrained refinement
*R*[*F*^2^ > 2σ(*F*^2^)] = 0.031	*w* = 1/[σ^2^(*F*_o_^2^) + (0.0448*P*)^2^ + 0.2292*P*] where *P* = (*F*_o_^2^ + 2*F*_c_^2^)/3
*wR*(*F*^2^) = 0.079	(Δ/σ)_max_ < 0.001
*S* = 1.06	Δρ_max_ = 0.20 e Å^−^^3^
7256 reflections	Δρ_min_ = −0.24 e Å^−^^3^
581 parameters	Absolute structure: Flack *x* determined using 3110 quotients [(*I*^+^)-(*I*^-^)]/[(*I*^+^)+(*I*^-^)] (Parsons *et al.*, 2013)
253 restraints	Absolute structure parameter: 0.00 (5)
Primary atom site location: dual	

### 5,10-Dihydroxy-9-methoxy-2,2-dimethyl-12-(2-methylbut-3-en-2-yl)-2H,6H-pyrano[3,2-b]xanthen-6-one Special details

**Table:**

Geometry. All esds (except the esd in the dihedral angle between two l.s. planes) are estimated using the full covariance matrix. The cell esds are taken into account individually in the estimation of esds in distances, angles and torsion angles; correlations between esds in cell parameters are only used when they are defined by crystal symmetry. An approximate (isotropic) treatment of cell esds is used for estimating esds involving l.s. planes.

### 5,10-Dihydroxy-9-methoxy-2,2-dimethyl-12-(2-methylbut-3-en-2-yl)-2H,6H-pyrano[3,2-b]xanthen-6-one Fractional atomic coordinates and isotropic or equivalent isotropic displacement parameters (Å^2^)

**Table:**

	*x*	*y*	*z*	*U*_iso_*/*U*_eq_	Occ. (<1)
O1	0.48934 (14)	0.63254 (10)	0.88990 (12)	0.0157 (3)	
C2	0.56134 (19)	0.68143 (15)	0.97693 (17)	0.0171 (4)	
C3	0.5962 (2)	0.77380 (14)	0.94037 (18)	0.0178 (4)	
H3	0.672019	0.801826	0.970704	0.021*	
C4	0.5213 (2)	0.81629 (15)	0.86586 (17)	0.0172 (4)	
H4	0.539570	0.876651	0.847378	0.021*	
C4A	0.4109 (2)	0.77029 (14)	0.81235 (17)	0.0153 (4)	
C5	0.3218 (2)	0.81468 (14)	0.74379 (16)	0.0151 (4)	
C6	0.13155 (19)	0.81253 (14)	0.61298 (16)	0.0153 (4)	
C6A	0.03222 (19)	0.75829 (14)	0.55740 (17)	0.0150 (4)	
C7	−0.0620 (2)	0.79472 (15)	0.48401 (16)	0.0174 (4)	
H7	−0.063051	0.857719	0.470036	0.021*	
C8	−0.1531 (2)	0.73982 (15)	0.43193 (17)	0.0179 (4)	
H8	−0.216283	0.764913	0.381753	0.021*	
C9	−0.15260 (19)	0.64708 (14)	0.45291 (16)	0.0159 (4)	
C10	−0.0607 (2)	0.60890 (14)	0.52692 (16)	0.0144 (4)	
C10A	0.03125 (19)	0.66544 (14)	0.57792 (16)	0.0141 (4)	
O11	0.11980 (13)	0.62404 (9)	0.64739 (12)	0.0151 (3)	
C11A	0.21661 (18)	0.67169 (14)	0.70155 (16)	0.0134 (4)	
C12	0.30334 (19)	0.62308 (14)	0.77021 (16)	0.0141 (4)	
C12A	0.39954 (19)	0.67621 (14)	0.82519 (16)	0.0137 (4)	
C13	0.6763 (2)	0.62260 (16)	1.00704 (18)	0.0211 (5)	
H13A	0.648844	0.563562	1.030848	0.032*	
H13B	0.731042	0.651523	1.064320	0.032*	
H13C	0.723247	0.614643	0.945423	0.032*	
C14	0.4786 (2)	0.68963 (16)	1.06809 (17)	0.0216 (5)	
H14A	0.399911	0.721312	1.043275	0.032*	
H14B	0.524359	0.723596	1.126368	0.032*	
H14C	0.458469	0.629226	1.092904	0.032*	
O15	0.33355 (14)	0.90468 (10)	0.73218 (12)	0.0197 (3)	
H15	0.275792	0.923517	0.687151	0.029*	
O16	0.14017 (14)	0.89588 (10)	0.59763 (12)	0.0206 (3)	
O17	−0.23789 (14)	0.58673 (10)	0.40592 (12)	0.0196 (3)	
C18	−0.3305 (2)	0.62000 (16)	0.32450 (18)	0.0217 (5)	
H18A	−0.287542	0.645740	0.266961	0.033*	
H18B	−0.381849	0.666699	0.353953	0.033*	
H18C	−0.385346	0.570258	0.296996	0.033*	
O19	−0.05738 (13)	0.51996 (10)	0.55172 (11)	0.0170 (3)	
H19	−0.098952	0.490660	0.503133	0.026*	
C5A	0.2222 (2)	0.76596 (15)	0.68675 (17)	0.0150 (4)	
C20	0.30221 (18)	0.51977 (14)	0.79184 (16)	0.0155 (4)	
C21	0.4241 (2)	0.47936 (15)	0.76007 (18)	0.0208 (4)	
H21	0.445444	0.494286	0.691477	0.025*	
C22	0.5035 (2)	0.42587 (17)	0.8174 (2)	0.0270 (5)	
H22A	0.577 (3)	0.404 (2)	0.790 (2)	0.036 (8)*	
H22B	0.495 (3)	0.413 (2)	0.890 (3)	0.044 (9)*	
C23	0.28615 (19)	0.50328 (14)	0.90934 (17)	0.0178 (4)	
H23A	0.279587	0.438304	0.922085	0.027*	
H23B	0.209051	0.533413	0.927059	0.027*	
H23C	0.359603	0.527643	0.953612	0.027*	
C24	0.1957 (2)	0.46614 (15)	0.72720 (19)	0.0239 (5)	
H24A	0.202985	0.473601	0.651468	0.036*	
H24B	0.113204	0.488743	0.743273	0.036*	
H24C	0.203356	0.402044	0.745839	0.036*	
O1_2	−0.00836 (14)	0.37560 (10)	0.13071 (12)	0.0194 (3)	
C2_2	−0.0698 (2)	0.33552 (15)	0.03272 (17)	0.0191 (4)	
C3_2	−0.0966 (2)	0.23753 (16)	0.05003 (18)	0.0205 (5)	
H3_2	−0.168920	0.209760	0.012961	0.025*	
C4_2	−0.0189 (2)	0.18992 (15)	0.11731 (17)	0.0188 (4)	
H4_2	−0.031187	0.126751	0.123982	0.023*	
C4A_2	0.08530 (19)	0.23455 (15)	0.18108 (17)	0.0162 (4)	
C5_2	0.17783 (19)	0.18563 (15)	0.24207 (16)	0.0157 (4)	
C5A_2	0.27205 (19)	0.23093 (15)	0.30899 (16)	0.0152 (4)	
C6_2	0.36855 (19)	0.18113 (14)	0.37328 (16)	0.0149 (4)	
C6A_2	0.46335 (19)	0.23343 (14)	0.43777 (17)	0.0155 (4)	
C7_2	0.5642 (2)	0.19459 (15)	0.50217 (17)	0.0171 (4)	
H7_2	0.573853	0.130840	0.503783	0.021*	
C8_2	0.6496 (2)	0.24795 (15)	0.56324 (17)	0.0179 (4)	
H8_2	0.717202	0.220815	0.607165	0.021*	
C9_2	0.6371 (2)	0.34219 (15)	0.56086 (17)	0.0166 (4)	
C10_2	0.5381 (2)	0.38304 (14)	0.49622 (16)	0.0149 (4)	
C10A_2	0.45264 (19)	0.32757 (14)	0.43611 (16)	0.0142 (4)	
O11_2	0.35792 (13)	0.37211 (9)	0.37575 (11)	0.0154 (3)	
C11A_2	0.26721 (19)	0.32619 (15)	0.31288 (16)	0.0149 (4)	
C12_2	0.17537 (19)	0.37960 (15)	0.25460 (17)	0.0174 (5)	
C12A_2	0.08667 (19)	0.32959 (14)	0.18777 (17)	0.0162 (4)	
C13_2	0.0193 (2)	0.34614 (17)	−0.05354 (18)	0.0243 (5)	
H13A_2	0.035750	0.410378	−0.064317	0.037*	
H13B_2	−0.020293	0.319796	−0.119763	0.037*	
H13C_2	0.099284	0.315045	−0.031762	0.037*	
C14_2	−0.1887 (2)	0.39184 (16)	0.00810 (19)	0.0231 (5)	
H14A_2	−0.242199	0.385921	0.065903	0.035*	
H14B_2	−0.235384	0.370661	−0.058027	0.035*	
H14C_2	−0.165275	0.455306	0.000533	0.035*	
O15_2	0.17522 (14)	0.09498 (10)	0.23611 (12)	0.0188 (3)	
H15_2	0.235733	0.073547	0.276462	0.028*	
O16_2	0.36869 (14)	0.09641 (10)	0.37326 (11)	0.0185 (3)	
O17_2	0.71448 (13)	0.40131 (10)	0.61783 (12)	0.0185 (3)	
C18_2	0.8165 (2)	0.36517 (16)	0.68843 (17)	0.0202 (5)	
H18A_2	0.873047	0.330205	0.647938	0.030*	
H18B_2	0.782263	0.325871	0.740454	0.030*	
H18C_2	0.864000	0.414731	0.725168	0.030*	
O19_2	0.52116 (14)	0.47341 (10)	0.48892 (12)	0.0197 (3)	
H19_2	0.570615	0.499177	0.535563	0.030*	
C20_2	0.1758 (2)	0.48402 (14)	0.26600 (18)	0.0209 (5)	
C21_2	0.0906 (4)	0.5329 (3)	0.1751 (4)	0.0166 (8)	0.5
H21_2	0.101463	0.518775	0.103741	0.020*	0.5
C21B_2	0.0423 (5)	0.5244 (3)	0.2329 (5)	0.0271 (11)	0.5
H21B_2	−0.028598	0.496895	0.259544	0.032*	0.5
C22_2	0.004 (2)	0.5934 (16)	0.1942 (15)	0.030 (3)	0.5
H22A_2	−0.008289	0.608386	0.265074	0.037*	0.5
H22B_2	−0.046065	0.621701	0.136995	0.037*	0.5
C22B_2	0.022 (2)	0.5936 (15)	0.1710 (15)	0.045 (4)	0.5
H22C_2	0.091559	0.622425	0.143254	0.054*	0.5
H22D_2	−0.061284	0.615457	0.153394	0.054*	0.5
C23_2	0.1694 (3)	0.51099 (16)	0.3812 (2)	0.0372 (6)	
H23A_2	0.095065	0.483015	0.407163	0.056*	
H23B_2	0.162826	0.576792	0.386166	0.056*	
H23C_2	0.246221	0.490474	0.424207	0.056*	
C24_2	0.2965 (3)	0.52333 (17)	0.2285 (2)	0.0374 (7)	
H24A_2	0.370556	0.499658	0.272679	0.056*	
H24B_2	0.294861	0.589264	0.234377	0.056*	
H24C_2	0.301451	0.506234	0.154607	0.056*	

### 5,10-Dihydroxy-9-methoxy-2,2-dimethyl-12-(2-methylbut-3-en-2-yl)-2H,6H-pyrano[3,2-b]xanthen-6-one Atomic displacement parameters (Å^2^)

**Table:**

	*U*^11^	*U*^22^	*U*^33^	*U*^12^	*U*^13^	*U*^23^
O1	0.0154 (7)	0.0140 (7)	0.0165 (7)	0.0001 (5)	−0.0043 (6)	0.0001 (6)
C2	0.0139 (10)	0.0208 (11)	0.0155 (10)	−0.0014 (8)	−0.0040 (8)	−0.0021 (8)
C3	0.0141 (10)	0.0176 (11)	0.0214 (11)	−0.0031 (8)	0.0008 (8)	−0.0047 (9)
C4	0.0164 (10)	0.0151 (10)	0.0204 (11)	−0.0022 (8)	0.0029 (8)	−0.0013 (8)
C4A	0.0165 (10)	0.0150 (11)	0.0147 (10)	−0.0012 (8)	0.0034 (8)	−0.0013 (8)
C5	0.0171 (11)	0.0126 (10)	0.0157 (10)	−0.0015 (8)	0.0027 (8)	0.0010 (8)
C6	0.0162 (11)	0.0160 (11)	0.0138 (10)	0.0002 (8)	0.0027 (8)	0.0009 (8)
C6A	0.0178 (11)	0.0136 (10)	0.0135 (10)	0.0000 (8)	0.0020 (8)	0.0008 (8)
C7	0.0199 (11)	0.0154 (10)	0.0166 (10)	0.0013 (8)	0.0007 (8)	0.0027 (8)
C8	0.0174 (10)	0.0190 (11)	0.0166 (10)	0.0039 (9)	−0.0012 (8)	0.0022 (9)
C9	0.0146 (10)	0.0190 (11)	0.0137 (10)	−0.0003 (8)	0.0006 (8)	−0.0023 (8)
C10	0.0185 (10)	0.0124 (10)	0.0125 (9)	−0.0001 (8)	0.0031 (8)	0.0003 (8)
C10A	0.0149 (10)	0.0170 (10)	0.0103 (9)	0.0039 (8)	0.0008 (8)	0.0025 (8)
O11	0.0162 (7)	0.0119 (7)	0.0159 (7)	−0.0006 (5)	−0.0044 (6)	0.0023 (6)
C11A	0.0132 (10)	0.0150 (10)	0.0120 (10)	−0.0001 (7)	0.0014 (8)	−0.0006 (8)
C12	0.0176 (10)	0.0123 (11)	0.0127 (10)	−0.0002 (7)	0.0033 (8)	0.0006 (8)
C12A	0.0136 (10)	0.0156 (10)	0.0120 (10)	0.0009 (8)	0.0019 (8)	0.0003 (8)
C13	0.0175 (11)	0.0241 (12)	0.0207 (11)	0.0009 (8)	−0.0031 (8)	0.0003 (9)
C14	0.0208 (11)	0.0247 (12)	0.0189 (11)	0.0000 (9)	0.0003 (8)	−0.0001 (9)
O15	0.0224 (8)	0.0119 (7)	0.0229 (8)	−0.0022 (6)	−0.0056 (6)	0.0038 (6)
O16	0.0248 (8)	0.0127 (8)	0.0229 (8)	−0.0007 (6)	−0.0039 (6)	0.0046 (6)
O17	0.0195 (7)	0.0172 (8)	0.0200 (7)	−0.0002 (6)	−0.0074 (6)	−0.0005 (6)
C18	0.0191 (11)	0.0229 (12)	0.0212 (11)	0.0021 (9)	−0.0071 (9)	−0.0022 (9)
O19	0.0199 (7)	0.0125 (7)	0.0172 (7)	−0.0009 (6)	−0.0048 (6)	−0.0003 (6)
C5A	0.0163 (10)	0.0147 (11)	0.0142 (10)	0.0002 (8)	0.0029 (8)	0.0015 (8)
C20	0.0166 (10)	0.0115 (10)	0.0176 (10)	0.0005 (8)	−0.0019 (8)	0.0023 (8)
C21	0.0263 (11)	0.0154 (10)	0.0214 (11)	−0.0004 (8)	0.0057 (9)	0.0002 (9)
C22	0.0248 (12)	0.0244 (12)	0.0327 (14)	0.0077 (10)	0.0062 (10)	0.0020 (10)
C23	0.0170 (10)	0.0157 (10)	0.0208 (10)	0.0014 (8)	0.0020 (8)	0.0043 (8)
C24	0.0288 (12)	0.0119 (10)	0.0282 (12)	−0.0028 (9)	−0.0093 (9)	0.0043 (9)
O1_2	0.0175 (7)	0.0180 (8)	0.0207 (8)	0.0011 (6)	−0.0062 (6)	0.0006 (6)
C2_2	0.0154 (10)	0.0232 (11)	0.0174 (10)	−0.0022 (8)	−0.0036 (8)	0.0022 (9)
C3_2	0.0146 (10)	0.0245 (11)	0.0215 (11)	−0.0059 (9)	−0.0023 (8)	−0.0008 (9)
C4_2	0.0168 (10)	0.0176 (11)	0.0217 (11)	−0.0039 (8)	0.0010 (8)	0.0006 (9)
C4A_2	0.0149 (10)	0.0192 (11)	0.0145 (10)	−0.0037 (8)	0.0020 (8)	−0.0002 (8)
C5_2	0.0180 (11)	0.0136 (10)	0.0157 (10)	−0.0024 (8)	0.0034 (8)	0.0014 (8)
C5A_2	0.0165 (10)	0.0157 (11)	0.0135 (10)	−0.0008 (8)	0.0019 (8)	0.0006 (8)
C6_2	0.0175 (10)	0.0141 (10)	0.0136 (10)	−0.0001 (8)	0.0039 (8)	0.0006 (8)
C6A_2	0.0167 (10)	0.0161 (11)	0.0139 (10)	0.0011 (8)	0.0027 (8)	0.0007 (8)
C7_2	0.0183 (10)	0.0139 (10)	0.0187 (11)	0.0012 (8)	0.0002 (8)	0.0025 (8)
C8_2	0.0166 (10)	0.0177 (11)	0.0186 (11)	0.0033 (8)	−0.0022 (8)	0.0031 (9)
C9_2	0.0153 (10)	0.0190 (11)	0.0152 (10)	−0.0025 (8)	0.0005 (8)	−0.0016 (8)
C10_2	0.0180 (10)	0.0124 (10)	0.0143 (10)	0.0006 (8)	0.0018 (8)	0.0009 (8)
C10A_2	0.0150 (10)	0.0155 (10)	0.0122 (10)	0.0021 (8)	0.0010 (8)	0.0020 (8)
O11_2	0.0163 (7)	0.0126 (7)	0.0162 (7)	0.0004 (5)	−0.0044 (6)	−0.0002 (6)
C11A_2	0.0157 (11)	0.0139 (10)	0.0149 (10)	−0.0022 (8)	0.0004 (8)	−0.0009 (8)
C12_2	0.0167 (10)	0.0133 (11)	0.0212 (11)	0.0010 (8)	−0.0020 (9)	0.0003 (8)
C12A_2	0.0143 (10)	0.0178 (11)	0.0160 (10)	0.0023 (8)	−0.0002 (8)	0.0028 (8)
C13_2	0.0185 (11)	0.0297 (13)	0.0241 (12)	−0.0011 (9)	−0.0007 (9)	0.0055 (10)
C14_2	0.0175 (10)	0.0281 (13)	0.0227 (11)	0.0018 (9)	−0.0030 (9)	0.0050 (10)
O15_2	0.0218 (8)	0.0120 (7)	0.0212 (8)	−0.0009 (6)	−0.0042 (6)	0.0014 (6)
O16_2	0.0227 (8)	0.0118 (7)	0.0199 (7)	0.0007 (6)	−0.0029 (6)	0.0018 (6)
O17_2	0.0183 (7)	0.0152 (7)	0.0198 (8)	0.0003 (6)	−0.0072 (6)	−0.0008 (6)
C18_2	0.0170 (10)	0.0211 (11)	0.0207 (11)	0.0010 (8)	−0.0067 (8)	0.0006 (9)
O19_2	0.0236 (8)	0.0113 (7)	0.0219 (8)	0.0003 (6)	−0.0083 (6)	−0.0015 (6)
C20_2	0.0211 (11)	0.0114 (10)	0.0281 (12)	0.0010 (8)	−0.0068 (9)	−0.0004 (9)
C21_2	0.021 (2)	0.010 (2)	0.018 (2)	−0.0030 (18)	0.0004 (18)	0.0042 (16)
C21B_2	0.022 (2)	0.019 (2)	0.038 (3)	0.0019 (19)	−0.008 (2)	−0.002 (2)
C22_2	0.028 (4)	0.027 (5)	0.035 (5)	0.012 (3)	−0.003 (4)	−0.001 (4)
C22B_2	0.046 (10)	0.022 (5)	0.059 (10)	0.004 (5)	−0.027 (7)	0.006 (6)
C23_2	0.0562 (16)	0.0155 (11)	0.0456 (16)	0.0025 (11)	0.0319 (13)	−0.0015 (11)
C24_2	0.0649 (18)	0.0137 (11)	0.0394 (15)	−0.0023 (11)	0.0315 (14)	0.0024 (10)

### 5,10-Dihydroxy-9-methoxy-2,2-dimethyl-12-(2-methylbut-3-en-2-yl)-2H,6H-pyrano[3,2-b]xanthen-6-one Geometric parameters (Å, º)

**Table:**

O1—C2	1.466 (2)	C2_2—C13_2	1.531 (3)
O1—C12A	1.355 (2)	C2_2—C14_2	1.518 (3)
C2—C3	1.504 (3)	C3_2—H3_2	0.9500
C2—C13	1.515 (3)	C3_2—C4_2	1.325 (3)
C2—C14	1.533 (3)	C4_2—H4_2	0.9500
C3—H3	0.9500	C4_2—C4A_2	1.458 (3)
C3—C4	1.328 (3)	C4A_2—C5_2	1.388 (3)
C4—H4	0.9500	C4A_2—C12A_2	1.411 (3)
C4—C4A	1.459 (3)	C5_2—C5A_2	1.411 (3)
C4A—C5	1.382 (3)	C5_2—O15_2	1.345 (3)
C4A—C12A	1.410 (3)	C5A_2—C6_2	1.443 (3)
C5—O15	1.349 (3)	C5A_2—C11A_2	1.413 (3)
C5—C5A	1.414 (3)	C6_2—C6A_2	1.452 (3)
C6—C6A	1.448 (3)	C6_2—O16_2	1.255 (3)
C6—O16	1.255 (3)	C6A_2—C7_2	1.399 (3)
C6—C5A	1.445 (3)	C6A_2—C10A_2	1.399 (3)
C6A—C7	1.400 (3)	C7_2—H7_2	0.9500
C6A—C10A	1.401 (3)	C7_2—C8_2	1.377 (3)
C7—H7	0.9500	C8_2—H8_2	0.9500
C7—C8	1.378 (3)	C8_2—C9_2	1.403 (3)
C8—H8	0.9500	C9_2—C10_2	1.400 (3)
C8—C9	1.400 (3)	C9_2—O17_2	1.356 (3)
C9—C10	1.399 (3)	C10_2—C10A_2	1.390 (3)
C9—O17	1.364 (3)	C10_2—O19_2	1.353 (3)
C10—C10A	1.393 (3)	C10A_2—O11_2	1.367 (2)
C10—O19	1.354 (3)	O11_2—C11A_2	1.365 (2)
C10A—O11	1.364 (2)	C11A_2—C12_2	1.403 (3)
O11—C11A	1.370 (2)	C12_2—C12A_2	1.409 (3)
C11A—C12	1.398 (3)	C12_2—C20_2	1.554 (3)
C11A—C5A	1.411 (3)	C13_2—H13A_2	0.9800
C12—C12A	1.413 (3)	C13_2—H13B_2	0.9800
C12—C20	1.555 (3)	C13_2—H13C_2	0.9800
C13—H13A	0.9800	C14_2—H14A_2	0.9800
C13—H13B	0.9800	C14_2—H14B_2	0.9800
C13—H13C	0.9800	C14_2—H14C_2	0.9800
C14—H14A	0.9800	O15_2—H15_2	0.8400
C14—H14B	0.9800	O17_2—C18_2	1.434 (2)
C14—H14C	0.9800	C18_2—H18A_2	0.9800
O15—H15	0.8400	C18_2—H18B_2	0.9800
O17—C18	1.435 (3)	C18_2—H18C_2	0.9800
C18—H18A	0.9800	O19_2—H19_2	0.8400
C18—H18B	0.9800	C20_2—C21_2	1.566 (5)
C18—H18C	0.9800	C20_2—C21B_2	1.555 (5)
O19—H19	0.8400	C20_2—C23_2	1.526 (3)
C20—C21	1.520 (3)	C20_2—C24_2	1.530 (3)
C20—C23	1.540 (3)	C21_2—H21_2	0.9500
C20—C24	1.544 (3)	C21_2—C22_2	1.33 (2)
C21—H21	0.9500	C21B_2—H21B_2	0.9500
C21—C22	1.317 (3)	C21B_2—C22B_2	1.29 (2)
C22—H22A	0.95 (3)	C22_2—H22A_2	0.9500
C22—H22B	0.95 (3)	C22_2—H22B_2	0.9500
C23—H23A	0.9800	C22B_2—H22C_2	0.9500
C23—H23B	0.9800	C22B_2—H22D_2	0.9500
C23—H23C	0.9800	C23_2—H23A_2	0.9800
C24—H24A	0.9800	C23_2—H23B_2	0.9800
C24—H24B	0.9800	C23_2—H23C_2	0.9800
C24—H24C	0.9800	C24_2—H24A_2	0.9800
O1_2—C2_2	1.467 (3)	C24_2—H24B_2	0.9800
O1_2—C12A_2	1.360 (2)	C24_2—H24C_2	0.9800
C2_2—C3_2	1.500 (3)		
			
C12A—O1—C2	119.84 (16)	C3_2—C2_2—C14_2	113.27 (18)
O1—C2—C3	110.01 (17)	C14_2—C2_2—C13_2	111.13 (19)
O1—C2—C13	104.27 (17)	C2_2—C3_2—H3_2	120.2
O1—C2—C14	108.31 (17)	C4_2—C3_2—C2_2	119.6 (2)
C3—C2—C13	112.53 (18)	C4_2—C3_2—H3_2	120.2
C3—C2—C14	109.97 (18)	C3_2—C4_2—H4_2	120.0
C13—C2—C14	111.53 (18)	C3_2—C4_2—C4A_2	120.0 (2)
C2—C3—H3	119.9	C4A_2—C4_2—H4_2	120.0
C4—C3—C2	120.21 (19)	C5_2—C4A_2—C4_2	121.5 (2)
C4—C3—H3	119.9	C5_2—C4A_2—C12A_2	119.04 (19)
C3—C4—H4	120.0	C12A_2—C4A_2—C4_2	119.2 (2)
C3—C4—C4A	120.0 (2)	C4A_2—C5_2—C5A_2	120.08 (19)
C4A—C4—H4	120.0	O15_2—C5_2—C4A_2	118.74 (18)
C5—C4A—C4	122.18 (19)	O15_2—C5_2—C5A_2	121.18 (19)
C5—C4A—C12A	118.84 (19)	C5_2—C5A_2—C6_2	120.81 (19)
C12A—C4A—C4	118.82 (19)	C5_2—C5A_2—C11A_2	118.03 (19)
C4A—C5—C5A	120.23 (19)	C11A_2—C5A_2—C6_2	121.15 (19)
O15—C5—C4A	118.31 (19)	C5A_2—C6_2—C6A_2	116.97 (18)
O15—C5—C5A	121.45 (19)	O16_2—C6_2—C5A_2	120.81 (19)
O16—C6—C6A	122.13 (19)	O16_2—C6_2—C6A_2	122.22 (19)
O16—C6—C5A	121.07 (19)	C7_2—C6A_2—C6_2	123.42 (19)
C5A—C6—C6A	116.79 (18)	C10A_2—C6A_2—C6_2	118.25 (19)
C7—C6A—C6	122.78 (19)	C10A_2—C6A_2—C7_2	118.3 (2)
C7—C6A—C10A	118.93 (19)	C6A_2—C7_2—H7_2	119.7
C10A—C6A—C6	118.30 (18)	C8_2—C7_2—C6A_2	120.6 (2)
C6A—C7—H7	119.8	C8_2—C7_2—H7_2	119.7
C8—C7—C6A	120.4 (2)	C7_2—C8_2—H8_2	119.9
C8—C7—H7	119.8	C7_2—C8_2—C9_2	120.3 (2)
C7—C8—H8	120.0	C9_2—C8_2—H8_2	119.9
C7—C8—C9	120.0 (2)	C10_2—C9_2—C8_2	120.43 (19)
C9—C8—H8	120.0	O17_2—C9_2—C8_2	125.52 (19)
C10—C9—C8	120.86 (19)	O17_2—C9_2—C10_2	114.05 (18)
O17—C9—C8	124.86 (19)	C10A_2—C10_2—C9_2	118.09 (19)
O17—C9—C10	114.27 (18)	O19_2—C10_2—C9_2	123.63 (19)
C10A—C10—C9	118.21 (18)	O19_2—C10_2—C10A_2	118.28 (18)
O19—C10—C9	123.27 (19)	C10_2—C10A_2—C6A_2	122.27 (19)
O19—C10—C10A	118.52 (18)	O11_2—C10A_2—C6A_2	122.90 (19)
C10—C10A—C6A	121.54 (18)	O11_2—C10A_2—C10_2	114.82 (18)
O11—C10A—C6A	122.95 (18)	C11A_2—O11_2—C10A_2	121.19 (17)
O11—C10A—C10	115.51 (18)	O11_2—C11A_2—C5A_2	119.48 (18)
C10A—O11—C11A	121.40 (16)	O11_2—C11A_2—C12_2	115.73 (18)
O11—C11A—C12	117.14 (18)	C12_2—C11A_2—C5A_2	124.78 (19)
O11—C11A—C5A	118.90 (18)	C11A_2—C12_2—C12A_2	113.75 (19)
C12—C11A—C5A	123.95 (19)	C11A_2—C12_2—C20_2	121.16 (18)
C11A—C12—C12A	114.45 (18)	C12A_2—C12_2—C20_2	125.09 (19)
C11A—C12—C20	126.87 (18)	O1_2—C12A_2—C4A_2	117.76 (19)
C12A—C12—C20	118.67 (18)	O1_2—C12A_2—C12_2	117.83 (19)
O1—C12A—C4A	118.63 (18)	C12_2—C12A_2—C4A_2	124.3 (2)
O1—C12A—C12	117.26 (18)	C2_2—C13_2—H13A_2	109.5
C4A—C12A—C12	124.03 (19)	C2_2—C13_2—H13B_2	109.5
C2—C13—H13A	109.5	C2_2—C13_2—H13C_2	109.5
C2—C13—H13B	109.5	H13A_2—C13_2—H13B_2	109.5
C2—C13—H13C	109.5	H13A_2—C13_2—H13C_2	109.5
H13A—C13—H13B	109.5	H13B_2—C13_2—H13C_2	109.5
H13A—C13—H13C	109.5	C2_2—C14_2—H14A_2	109.5
H13B—C13—H13C	109.5	C2_2—C14_2—H14B_2	109.5
C2—C14—H14A	109.5	C2_2—C14_2—H14C_2	109.5
C2—C14—H14B	109.5	H14A_2—C14_2—H14B_2	109.5
C2—C14—H14C	109.5	H14A_2—C14_2—H14C_2	109.5
H14A—C14—H14B	109.5	H14B_2—C14_2—H14C_2	109.5
H14A—C14—H14C	109.5	C5_2—O15_2—H15_2	109.5
H14B—C14—H14C	109.5	C9_2—O17_2—C18_2	117.80 (17)
C5—O15—H15	109.5	O17_2—C18_2—H18A_2	109.5
C9—O17—C18	117.48 (16)	O17_2—C18_2—H18B_2	109.5
O17—C18—H18A	109.5	O17_2—C18_2—H18C_2	109.5
O17—C18—H18B	109.5	H18A_2—C18_2—H18B_2	109.5
O17—C18—H18C	109.5	H18A_2—C18_2—H18C_2	109.5
H18A—C18—H18B	109.5	H18B_2—C18_2—H18C_2	109.5
H18A—C18—H18C	109.5	C10_2—O19_2—H19_2	109.5
H18B—C18—H18C	109.5	C12_2—C20_2—C21_2	113.4 (2)
C10—O19—H19	109.5	C12_2—C20_2—C21B_2	111.4 (2)
C5—C5A—C6	119.86 (19)	C23_2—C20_2—C12_2	110.50 (19)
C11A—C5A—C5	118.49 (19)	C23_2—C20_2—C21_2	120.3 (2)
C11A—C5A—C6	121.62 (19)	C23_2—C20_2—C21B_2	91.4 (3)
C21—C20—C12	108.51 (16)	C23_2—C20_2—C24_2	108.9 (2)
C21—C20—C23	112.43 (17)	C24_2—C20_2—C12_2	110.11 (18)
C21—C20—C24	104.60 (18)	C24_2—C20_2—C21_2	91.8 (2)
C23—C20—C12	109.34 (16)	C24_2—C20_2—C21B_2	122.7 (3)
C23—C20—C24	106.41 (17)	C20_2—C21_2—H21_2	118.8
C24—C20—C12	115.57 (17)	C22_2—C21_2—C20_2	122.4 (9)
C20—C21—H21	116.5	C22_2—C21_2—H21_2	118.8
C22—C21—C20	127.0 (2)	C20_2—C21B_2—H21B_2	118.2
C22—C21—H21	116.5	C22B_2—C21B_2—C20_2	123.6 (12)
C21—C22—H22A	120.5 (18)	C22B_2—C21B_2—H21B_2	118.2
C21—C22—H22B	122.2 (19)	C21_2—C22_2—H22A_2	120.0
H22A—C22—H22B	117 (3)	C21_2—C22_2—H22B_2	120.0
C20—C23—H23A	109.5	H22A_2—C22_2—H22B_2	120.0
C20—C23—H23B	109.5	C21B_2—C22B_2—H22C_2	120.0
C20—C23—H23C	109.5	C21B_2—C22B_2—H22D_2	120.0
H23A—C23—H23B	109.5	H22C_2—C22B_2—H22D_2	120.0
H23A—C23—H23C	109.5	C20_2—C23_2—H23A_2	109.5
H23B—C23—H23C	109.5	C20_2—C23_2—H23B_2	109.5
C20—C24—H24A	109.5	C20_2—C23_2—H23C_2	109.5
C20—C24—H24B	109.5	H23A_2—C23_2—H23B_2	109.5
C20—C24—H24C	109.5	H23A_2—C23_2—H23C_2	109.5
H24A—C24—H24B	109.5	H23B_2—C23_2—H23C_2	109.5
H24A—C24—H24C	109.5	C20_2—C24_2—H24A_2	109.5
H24B—C24—H24C	109.5	C20_2—C24_2—H24B_2	109.5
C12A_2—O1_2—C2_2	119.05 (16)	C20_2—C24_2—H24C_2	109.5
O1_2—C2_2—C3_2	109.96 (18)	H24A_2—C24_2—H24B_2	109.5
O1_2—C2_2—C13_2	108.29 (17)	H24A_2—C24_2—H24C_2	109.5
O1_2—C2_2—C14_2	103.65 (17)	H24B_2—C24_2—H24C_2	109.5
C3_2—C2_2—C13_2	110.26 (19)		
			
O1—C2—C3—C4	30.5 (3)	C2_2—C3_2—C4_2—C4A_2	5.5 (3)
C2—O1—C12A—C4A	26.1 (3)	C3_2—C4_2—C4A_2—C5_2	−171.6 (2)
C2—O1—C12A—C12	−157.12 (17)	C3_2—C4_2—C4A_2—C12A_2	13.6 (3)
C2—C3—C4—C4A	−5.6 (3)	C4_2—C4A_2—C5_2—C5A_2	−175.24 (18)
C3—C4—C4A—C5	171.99 (19)	C4_2—C4A_2—C5_2—O15_2	4.6 (3)
C3—C4—C4A—C12A	−12.6 (3)	C4_2—C4A_2—C12A_2—O1_2	−2.2 (3)
C4—C4A—C5—O15	−4.6 (3)	C4_2—C4A_2—C12A_2—C12_2	173.6 (2)
C4—C4A—C5—C5A	174.56 (18)	C4A_2—C5_2—C5A_2—C6_2	179.70 (18)
C4—C4A—C12A—O1	2.4 (3)	C4A_2—C5_2—C5A_2—C11A_2	1.1 (3)
C4—C4A—C12A—C12	−174.12 (19)	C5_2—C4A_2—C12A_2—O1_2	−177.14 (18)
C4A—C5—C5A—C6	−178.24 (18)	C5_2—C4A_2—C12A_2—C12_2	−1.3 (3)
C4A—C5—C5A—C11A	−0.1 (3)	C5_2—C5A_2—C6_2—C6A_2	178.57 (18)
C5—C4A—C12A—O1	177.93 (18)	C5_2—C5A_2—C6_2—O16_2	−2.0 (3)
C5—C4A—C12A—C12	1.4 (3)	C5_2—C5A_2—C11A_2—O11_2	−179.13 (17)
C6—C6A—C7—C8	178.87 (18)	C5_2—C5A_2—C11A_2—C12_2	−0.2 (3)
C6—C6A—C10A—C10	−179.52 (18)	C5A_2—C6_2—C6A_2—C7_2	−178.49 (19)
C6—C6A—C10A—O11	−0.3 (3)	C5A_2—C6_2—C6A_2—C10A_2	2.1 (3)
C6A—C6—C5A—C5	180.00 (17)	C5A_2—C11A_2—C12_2—C12A_2	−1.3 (3)
C6A—C6—C5A—C11A	1.9 (3)	C5A_2—C11A_2—C12_2—C20_2	178.5 (2)
C6A—C7—C8—C9	0.6 (3)	C6_2—C5A_2—C11A_2—O11_2	2.3 (3)
C6A—C10A—O11—C11A	−0.3 (3)	C6_2—C5A_2—C11A_2—C12_2	−178.76 (19)
C7—C6A—C10A—C10	0.2 (3)	C6_2—C6A_2—C7_2—C8_2	−178.62 (18)
C7—C6A—C10A—O11	179.41 (18)	C6_2—C6A_2—C10A_2—C10_2	179.21 (18)
C7—C8—C9—C10	0.3 (3)	C6_2—C6A_2—C10A_2—O11_2	−0.7 (3)
C7—C8—C9—O17	−179.88 (18)	C6A_2—C7_2—C8_2—C9_2	−0.6 (3)
C8—C9—C10—C10A	−1.0 (3)	C6A_2—C10A_2—O11_2—C11A_2	0.0 (3)
C8—C9—C10—O19	178.67 (19)	C7_2—C6A_2—C10A_2—C10_2	−0.2 (3)
C8—C9—O17—C18	3.8 (3)	C7_2—C6A_2—C10A_2—O11_2	179.82 (18)
C9—C10—C10A—C6A	0.7 (3)	C7_2—C8_2—C9_2—C10_2	−0.2 (3)
C9—C10—C10A—O11	−178.59 (17)	C7_2—C8_2—C9_2—O17_2	179.52 (19)
C10—C9—O17—C18	−176.38 (17)	C8_2—C9_2—C10_2—C10A_2	0.7 (3)
C10—C10A—O11—C11A	178.99 (16)	C8_2—C9_2—C10_2—O19_2	−179.11 (19)
C10A—C6A—C7—C8	−0.8 (3)	C8_2—C9_2—O17_2—C18_2	−1.4 (3)
C10A—O11—C11A—C12	−179.40 (16)	C9_2—C10_2—C10A_2—C6A_2	−0.5 (3)
C10A—O11—C11A—C5A	1.7 (3)	C9_2—C10_2—C10A_2—O11_2	179.42 (17)
O11—C11A—C12—C12A	−178.88 (16)	C10_2—C9_2—O17_2—C18_2	178.34 (17)
O11—C11A—C12—C20	0.2 (3)	C10_2—C10A_2—O11_2—C11A_2	−179.90 (16)
O11—C11A—C5A—C5	179.38 (17)	C10A_2—C6A_2—C7_2—C8_2	0.8 (3)
O11—C11A—C5A—C6	−2.5 (3)	C10A_2—O11_2—C11A_2—C5A_2	−0.8 (3)
C11A—C12—C12A—O1	−177.53 (16)	C10A_2—O11_2—C11A_2—C12_2	−179.85 (17)
C11A—C12—C12A—C4A	−1.0 (3)	O11_2—C11A_2—C12_2—C12A_2	177.64 (17)
C11A—C12—C20—C21	119.1 (2)	O11_2—C11A_2—C12_2—C20_2	−2.5 (3)
C11A—C12—C20—C23	−117.9 (2)	C11A_2—C5A_2—C6_2—C6A_2	−2.9 (3)
C11A—C12—C20—C24	2.1 (3)	C11A_2—C5A_2—C6_2—O16_2	176.54 (19)
C12—C11A—C5A—C5	0.5 (3)	C11A_2—C12_2—C12A_2—O1_2	177.95 (17)
C12—C11A—C5A—C6	178.64 (18)	C11A_2—C12_2—C12A_2—C4A_2	2.1 (3)
C12—C20—C21—C22	129.5 (2)	C11A_2—C12_2—C20_2—C21_2	164.8 (3)
C12A—O1—C2—C3	−41.4 (2)	C11A_2—C12_2—C20_2—C21B_2	−156.8 (3)
C12A—O1—C2—C13	−162.25 (16)	C11A_2—C12_2—C20_2—C23_2	−56.8 (3)
C12A—O1—C2—C14	78.9 (2)	C11A_2—C12_2—C20_2—C24_2	63.6 (3)
C12A—C4A—C5—O15	179.98 (18)	C12_2—C20_2—C21_2—C22_2	128.5 (13)
C12A—C4A—C5—C5A	−0.8 (3)	C12_2—C20_2—C21B_2—C22B_2	−133.2 (12)
C12A—C12—C20—C21	−61.8 (2)	C12A_2—O1_2—C2_2—C3_2	44.4 (2)
C12A—C12—C20—C23	61.1 (2)	C12A_2—O1_2—C2_2—C13_2	−76.1 (2)
C12A—C12—C20—C24	−178.89 (18)	C12A_2—O1_2—C2_2—C14_2	165.80 (17)
C13—C2—C3—C4	146.3 (2)	C12A_2—C4A_2—C5_2—C5A_2	−0.5 (3)
C14—C2—C3—C4	−88.7 (2)	C12A_2—C4A_2—C5_2—O15_2	179.34 (18)
O15—C5—C5A—C6	0.9 (3)	C12A_2—C12_2—C20_2—C21_2	−15.3 (4)
O15—C5—C5A—C11A	179.09 (18)	C12A_2—C12_2—C20_2—C21B_2	23.0 (4)
O16—C6—C6A—C7	−0.9 (3)	C12A_2—C12_2—C20_2—C23_2	123.1 (2)
O16—C6—C6A—C10A	178.77 (19)	C12A_2—C12_2—C20_2—C24_2	−116.5 (2)
O16—C6—C5A—C5	0.7 (3)	C13_2—C2_2—C3_2—C4_2	86.9 (3)
O16—C6—C5A—C11A	−177.38 (19)	C14_2—C2_2—C3_2—C4_2	−147.9 (2)
O17—C9—C10—C10A	179.24 (17)	O15_2—C5_2—C5A_2—C6_2	−0.1 (3)
O17—C9—C10—O19	−1.1 (3)	O15_2—C5_2—C5A_2—C11A_2	−178.65 (18)
O19—C10—C10A—C6A	−178.94 (18)	O16_2—C6_2—C6A_2—C7_2	2.1 (3)
O19—C10—C10A—O11	1.8 (3)	O16_2—C6_2—C6A_2—C10A_2	−177.35 (19)
C5A—C6—C6A—C7	179.82 (18)	O17_2—C9_2—C10_2—C10A_2	−179.01 (17)
C5A—C6—C6A—C10A	−0.5 (3)	O17_2—C9_2—C10_2—O19_2	1.1 (3)
C5A—C11A—C12—C12A	0.0 (3)	O19_2—C10_2—C10A_2—C6A_2	179.34 (19)
C5A—C11A—C12—C20	179.06 (19)	O19_2—C10_2—C10A_2—O11_2	−0.7 (3)
C20—C12—C12A—O1	3.3 (3)	C20_2—C12_2—C12A_2—O1_2	−1.9 (3)
C20—C12—C12A—C4A	179.87 (18)	C20_2—C12_2—C12A_2—C4A_2	−177.8 (2)
C23—C20—C21—C22	8.5 (3)	C23_2—C20_2—C21_2—C22_2	−5.5 (13)
C24—C20—C21—C22	−106.6 (3)	C23_2—C20_2—C21B_2—C22B_2	114.1 (12)
O1_2—C2_2—C3_2—C4_2	−32.4 (3)	C24_2—C20_2—C21_2—C22_2	−118.7 (13)
C2_2—O1_2—C12A_2—C4A_2	−28.2 (3)	C24_2—C20_2—C21B_2—C22B_2	0.5 (13)
C2_2—O1_2—C12A_2—C12_2	155.71 (18)		

### 5,10-Dihydroxy-9-methoxy-2,2-dimethyl-12-(2-methylbut-3-en-2-yl)-2H,6H-pyrano[3,2-b]xanthen-6-one Hydrogen-bond geometry (Å, º)

**Table:**

*D*—H···*A*	*D*—H	H···*A*	*D*···*A*	*D*—H···*A*
O15—H15···O16	0.84	1.78	2.530 (2)	147
O15_2—H15_2···O16_2	0.84	1.80	2.547 (2)	147
O19—H19···O16^i^	0.84	1.92	2.719 (2)	159
O19_2—H19_2···O16_2^ii^	0.84	1.92	2.704 (2)	156

Symmetry codes: (i) −*x*, *y*−1/2, −*z*+1; (ii) −*x*+1, *y*+1/2, −*z*+1.
